# EGFP标记的Lewis肺癌细胞构建裸鼠恶性胸腔积液模型

**DOI:** 10.3779/j.issn.1009-3419.2012.06.01

**Published:** 2012-06-20

**Authors:** 兴群 马, 宇 孙, 守巨 王, 志坚 杨, 勇 宋

**Affiliations:** 1 210002 南京，南京大学医学院临床学院，南京军区南京总医院呼吸内科 Department of Respiratory Medicine, Nanjing General Hospital of Nanjing Command, Clinical School of the Medical College of Nanjing University, Nanjing 210002, China; 2 210002 南京，南京源端生物科技有限公司 Nanjing Origin Center for Tumor Animal Model, Nanjing 210002, China; 3 210002 南京，南京大学医学院临床学院，南京军区南京总医院医学影像科 Department of Medical Imaging, Nanjing General Hospital of Nanjing Command, Clinical School of the Medical College of Nanjing University, Nanjing 210002, China

**Keywords:** 恶性胸腔积液, 增强型绿色荧光蛋白, 裸鼠模型, 积分光密度, Malignant pleural e?usion, Enhanced green ￡uorescent protein, Nude mouse model, Integrated optical density

## Abstract

**背景与目的:**

恶性胸腔积液是晚期肺癌预后差的因素之一。本研究拟用Lewis肺癌细胞构建裸鼠恶性胸腔积液模型，从而建立一个良好的动物实验研究平台。

**方法:**

经胸膜腔接种表达增强型绿色荧光蛋白的Lewis肺癌细胞，建立裸鼠恶性胸腔积液模型。定期解剖小鼠，经小动物活体荧光成像系统观察肿瘤的生长。剩余小鼠定期行胸部CT检查，计算各时间点的成胸水率，并观察建模后裸鼠的生存期和肿瘤转移情况。所有小鼠在解剖时发现有胸水，抽取并计量，同一时间点内获得多份胸水标本，计算其平均体积。利用相关性检验分析胸水体积与肿瘤积分光密度之间的相关性。

**结果:**

接种后第4天，荧光体视镜下可发现胸膜上有绿色荧光，成瘤率100%。随接种时间延长，肿瘤体积逐渐增加，肿瘤侵及纵隔和肺门淋巴结、对侧胸膜、心包，转移率分别为87%、73%和20%。第7天、第14天和第21天成胸水率分别为13%、46%和53%。小鼠平均生存时间为28.8天，所有胸水均为血性，胸水平均体积在第10天以后逐渐增加，第16天达到峰值（0.5 mL）。胸水体积与积分光密度之间具有相关性（*r*=0.91, *P* < 0.000, 1）。

**结论:**

本研究将表达增强型绿色荧光蛋白的肺癌细胞在显微镜下经胸膜腔接种成功建立肺癌恶性胸腔积液模型，有助于动态观察肿瘤细胞在胸腔内的生物学行为，该模型可应用于肺癌的基础研究及抗肿瘤药物开发。

## Abstract (en)

**Background and objective:**

Malignant pleural effusion (MPE) is a poor prognosis factor in patients with advanced lung cancer. The aim of this study is to establish a mouse model of MPE using Lewis lung carcinoma (LLC) cell lines expressing enhanced green fluorescent protein (EGFP).

**Methods:**

The mouse model was created by injecting LLC-EGFP cells directly into the pleural cavity of mice that were sacrificed periodically. The dynamic growth and metastasis of tumor cells were screened using *in vivo* fluorescence imaging. The remaining mice were subjected to transverse computed tomography (CT) imaging periodically to analyze the formation rate of pleural effusion. The survival rate and tumor metastasis were also observed. Pleural fluid was gently aspirated using a 1 mL syringe and its volume was measured. When two or more mice bore pleural effusion at the same time, we calculated the average volume. The correlation of pleural effusion with the integrated optical density (IOD) were analyzed.

**Results:**

Four days after the inoculation of LLC-EGFP cells, green fluorescence was observed by opening the chest wall. The tumor formation rate was 100%, and the IOD gradually increased after inoculation. The metastasis sites were mediastinal, and the hilar lymph nodes were contralateral pleural as well as pericardial. The metastasis rates were 87%, 73% and 20%, respectively. The CT scan revealed that the formation rates of pleural effusion on days 7, 14 and 21 were 13%, 46% and 53%, respectively. The average volume of pleural effusion increased obviously on day 10 and peaked on day 16 with a value of 0.5 mL. The mean survival time of nude mice was 28.8 days. The volume of pleural effusion and IOD were significantly correlated (*r*=0.91, *P* < 0.000, 1).

**Conclusion:**

A mouse model of lung cancer malignant pleural effusion was successfully established by injecting LLC lines expressing EGFP into the pleural cavity under a microscope. The model can enable dynamic observations of the biological behavior of tumor cells in the pleural cavity. It might be helpful for basic research on advanced lung cancer as well as anti-tumor drug development.

恶性胸腔积液（malignant pleural effusion, MPE）是晚期肺癌的常见死亡原因之一，临床治疗效果差，建立恶性胸腔积液模型具有积极意义。已报道^[1]^可利用经尾静脉、经气管内和经胸膜腔接种瘤细胞的方法建立MPE模型，但是经气道接种，手术相关死亡率高达17%，肿瘤定植在气道或者肺部，但很少累及胸膜，成胸水率低^[2]^，同一株肺癌细胞分别经尾静脉和经胸膜腔接种，后者成胸水率更高，胸水量更大，并能避免肿瘤细胞在多脏器转移^[3]^。经胸膜腔接种方法虽与肿瘤细胞转移到胸膜腔的生物学行为不尽相同，但是可以单独地研究肿瘤细胞在胸膜腔内的一系列生物学行为。绿色荧光蛋白（green fluorescnt protein, GFP）是一种非酶性报告基因，转染肿瘤细胞后，将随着肿瘤细胞的分裂、生长而传给下代，也将随着肿瘤细胞的死亡而消亡，在转基因及肿瘤活体成像研究中得到广泛应用^[4]^。活体荧光显像技术为直接观察肿瘤的发生、生长、转移、血管生成和肿瘤细胞与宿主微环境的相互作用等生物学行为及直接客观评价抗肿瘤药物疗效提供了重要的手段。迄今为止，国内尚无该技术用于MPE模型的报道。因此，本研究拟通过表达增强型绿色荧光蛋白的Lewis肺癌细胞（LLC-EGFP）经胸膜腔接种方法，建立裸鼠恶性胸腔积液模型，并应用小动物活体荧光成像系统观察肿瘤的生长，进一步探索其生物学特性，用于研究肺癌的胸膜转移和临床前期药物研发。

## 1 材料与方法

### 1.1 材料

#### 1.1.1 细胞系

LLC-EGFP：购自美国ATCC（AmericanType Culture Collection美国组织细胞库）。

#### 1.1.2 实验动物

BALB/c（nu/nu）裸鼠，35只雄性，5只雌性，4周龄-6周龄，体重19 g-27 g。所有裸鼠在SPF级屏障系统中饲养并进行实验（实验室使用许可证编号：SYXK(苏)2007-0011）。饲养室相对湿度为55%±10%，温度为22 ℃±2 ℃，光照12 h明暗交替，裸鼠饲养用的饲料为钴60辐射灭菌过的大小鼠专用颗粒饲料（江苏省协同医药生物工程有限公司）。

#### 1.1.3 仪器设备

西门子SENSATION 16获取CT图像，管电压120 kVp，电流93 A；动物活体荧光影像系统由体视显微镜（ZOOM645S，江南禹成光学仪器有限公司）；CDC（Retiga Exi cooled Digital Color型，美国QIMAGING公司）；荧光激发仪（LG-150-A型，南京超腾科技发展有限公司）组成。

### 1.2 方法

#### 1.2.1 LLC-EGFP细胞系

在浓度为10%的RPMI-1640培养液中37 ℃、5%CO_2_饱和湿度条件下培养，每3天传代1次，取生长良好细胞用于实验。

#### 1.2.2 恶性胸腔积液裸鼠模型的建立

将LLC-EGFP细胞悬浮于磷酸缓冲液（PBS）中，调整细胞浓度为5×10^5^/50 μL。氯胺酮配比液肌肉麻醉，小鼠仰卧位呈大字型固定，消毒心前区皮肤，于胸骨柄右侧0.5 cm处做一纵行切除，切口长0.5 cm，分离皮肤及皮下筋膜，露出肋间肌肉，棉签压迫止血。微量移液器抽吸50 μL细胞悬液，于显微镜下避开血管，经肋间隙注入胸膜腔，此过程中速度不宜过快，针尖注入深度约3 mm-5 mm，以免刺破脏层胸膜或者伤及肺部。注射完毕后缝合伤口，酒精棉球消毒，待裸鼠苏醒后安放于原饲养笼内，给予充分饲养和饮水。整个过程在无菌操作台上完成。

#### 1.2.3 监测指标

① 术后每天观察动物一般状态，包括进食、活动、外观、对外界刺激的反应。②观察肿瘤生长：接种后第4天开始，每隔3天随机麻醉处死3只动物，拟观察时间为3周。解剖荷瘤鼠，分别于自然光源和激发光源下拍照，使用小动物活体成像系统检测GFP的表达。③其余小鼠在发现GFP表达后第3天，行胸部CT检查，观察该时间点的胸水形成并计算成胸水率；第14天及第21天重复检查并计算成胸水率。发现胸水的小鼠在耳朵上做小切口以示标记，观察与无胸水小鼠的生存时间差异。当裸鼠出现恶病质后，解剖荷瘤鼠，肉眼观察肿瘤生长情况及周围脏器受累情况，并拍照。④所有小鼠解剖时先暴露腹腔，用1 mL注射器在双侧横隔下抽吸胸水，并计量，若同一时间获得多份胸水标本，计算其平均体积，胸水涂片行脱落细胞学检查。⑤取胸膜上肿瘤组织行HE染色。

#### 1.2.4 胸部CT扫描方法

小鼠麻醉后，呈仰卧位固定头部，探测器准直器64 mm×0.6 mm，机架旋转时间为0.5 s，螺距为1.4，管电压为120 kVp，参考管电流93 A，将扫描获得的图像实时传输到多功能图像后处理工作站（Syngo MMWP CTworkplaceVA30A）。

#### 1.2.5 拍照方法

充分暴露小鼠胸腔，使用小动物活体成像系统检测GFP的表达，发射波长为520 nm，激发波长为480 nm，曝光时间为1 s。

#### 1.2.6 HE染色方法和胸水图片制作方法

制作方法参考相关文献^[6]^。

#### 1.2.7 DT2000图像分析软件分析肿瘤积分光密度（intergral optical density, IOD）值

设置软件参数为周长和积分光密度，对胸腔内肿瘤结节进行手动测量周长，系统自动生成相对应的IOD值，并传到Excel表上，计算每只小鼠的IOD值总和，每只小鼠重复检测3次，取平均值。以接种时间为横坐标，取该时间点的胸水平均体积为纵坐标，绘制胸水形成曲线，并分析同一只小鼠的胸水量与IOD值的相关性。

### 1.3 统计学处理

采用SPSS 13.0进行统计学分析。相关性检验分析小鼠胸水量与其肿瘤积分光密度之间的相关性，以*P* < 0.05为差异具有统计学意义。

## 2 结果

### 2.1 活体荧光成像观察肿瘤生长

整个手术过程顺利，术中4只小鼠死亡，其余小鼠均完成实验。21只小鼠用于观察肿瘤生长。第4天胸壁上可见绿色荧光，第10天出现对侧胸膜、肺门和纵隔淋巴结转移，第16天转移灶体积明显增加，第25天肿瘤组织填满整个胸腔（图 1）。

**1:**
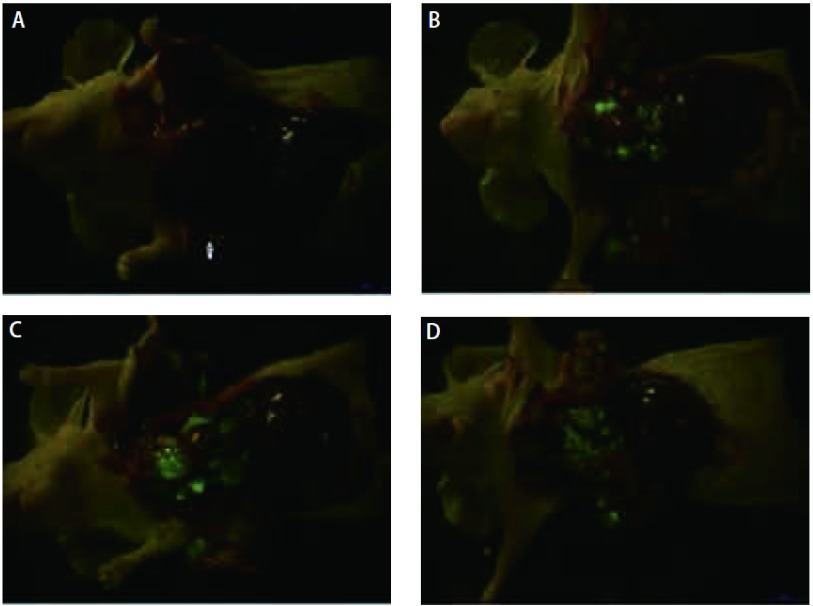
活体荧光成像动态观察肿瘤细胞接种后裸鼠胸腔内肿瘤生长。A：第4天右侧壁层胸膜上可见绿色荧光表达（箭头所示）；B：第10天肿瘤向对侧胸膜、纵膈和肺门淋巴结转移；C：第16天转移灶数量逐渐增多，体积逐渐增加；D：第25天肿瘤组织填满整个胸腔。 Dynamic observation of fluorescence imaging of tumor in the cavity of nude mice after LLC-EGFP cells inoculation. A: Green fluorescence was scaned in the right parietal pleural at day 4 (arrow); B: Contralateral pleural metastasis, mediastinal and hilar lymph node metastasis were found at day 10; C: Tumor volume and tumor foci were also obviously increased at day 16; D: The whole chest was entirely occupied at day 25.

### 2.2 成胸水率、生存时间和肿瘤转移

15只小鼠用于此部分实验，第7天可见2只小鼠有单侧胸腔积液，最早成胸水率为13%，第14天成胸水率为46%，第21天发现5只有双侧胸腔积液，3只有单侧积液，最高成胸水率为53%。图示为同一只小鼠分别在第7天、第14天和第21天的胸部CT检查（图 2）。壁层胸膜上肿瘤组织切片行HE染色，可见腺癌细胞聚集，胸水涂片染色提示LLC细胞核大，染色深（图 3）。小鼠平均生存期为28.8天，有胸水小鼠在第20天以后活动量明显下降，呼吸急促，皮肤变为苍白色，对外界刺激的反应差，相对无胸水小鼠，生存时间偏短（图 4）。解剖荷瘤鼠，大体观察可见小鼠的胸膜及纵隔内多发转移性肿瘤结节，成颗粒样聚集，颜色灰暗，少数瘤体出现破溃；荧光显微镜观察转移灶成像更加清晰，其中13只发生纵隔及肺门淋巴结转移，11只发生对侧胸膜转移，3只出现心包积液，转移率分别为87%、73%和20%。而肝脏、骨骼、肾上腺尚未出现转移（图 5）。

**2:**
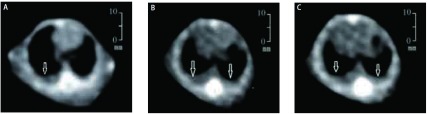
胸部CT连续观察同一只裸鼠胸腔积液形成。A：第7天见右侧胸腔积液；B：第14天出现双侧胸腔积液，体积较前增加；C：第21天见胸腔积液稍减少（箭头所示）。 Continuous observation of malignant pleural effusion via CT imagines on nude mise after LLC-EGFP cells inoculation. A: Malignant pleural effusion (MPE) was scaned in the right cavity at day 7 (arrow); B: A developing pleural effusion can be seen in the bilateral cavity at day 14 (arrows); C: The volume was slightly decreased at day 21 (arrows).

**3:**
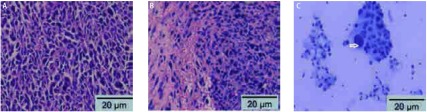
胸膜肿瘤的组织学检查和胸水脱落细胞学检查。A：胸膜上的肿瘤组织行HE染色，提示肿瘤由腺癌细胞构成；B：肿瘤侵润壁层胸膜；C：胸水涂片用改良型吉姆萨染色行脱落细胞检查，提示LLC细胞核大、深染（×200）。 Histology of pleural tumors and cytology of malignant pleural effusions in nude mice. A: Section through a small parietal pleural Lewis lung cancer implantation stained with hematoxylin and eosin shows that pleural tumors consisted of adenocarcinomatous cells; B: Tumors grew on the pleural surface. C: Representative photomicrograph of a cytospin from several specimens of malignant pleural effusion stained with modified Wright's-Giemsa stain, LLC cells with large nuclei and visible nucleoli (arrow)(×200).

**4:**
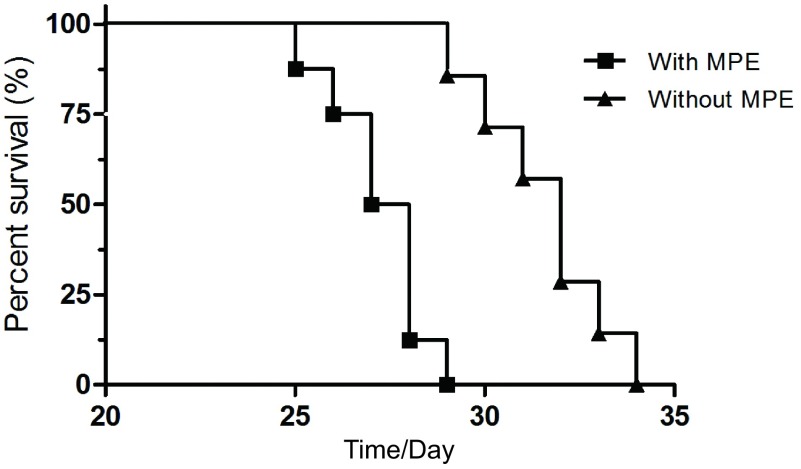
裸鼠生存时间曲线。15只小鼠分别在25天-31天内死亡，平均生存时间为28.8天。有胸水小鼠在20天以后出现消瘦，进食减少，呼吸急促，对外界刺激的反应差，生存时间较无胸水小鼠偏短。 Survival curve of nude mice. 15 mice died during 25 d -31 d and the average survival time was 28.8 d. Mice bearing pleural effusion appeared emaciated, loss of appetite, shortness of breath and insensitivity to external stimuli at 20 d after LLC-EGFP cells inoculation. The survival time was shorter compared to nude mice without pleural effusion.

**5:**
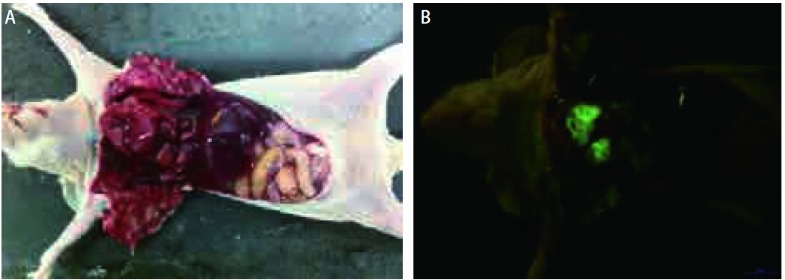
裸鼠自然死亡肿瘤的解剖观察。A：大体观察；B：荧光照片。整体荧光成像对微小转移灶显像比自然光下大体照片显像更为清晰。 Observation of nude mouse after thoracic anatomy. A: The general picture; B: Fluorescence imaging. Compared to the general picture, the fluorescence imaging is more clear to detect minor tumours.

### 2.3 胸腔积液形成曲线绘制，胸水体积与肿瘤积分光密度的相关性分析

36只小鼠完成实验，共17只小鼠有胸腔积液。第10天胸水平均体积逐渐增加，第16天达到高峰，最大量为0.5 mL，后期胸水量稍有下降。胸水形成曲线与影像学观察结果基本一致（图 6）。小鼠在荧光体视显微镜下拍照，经过后期DT2000图像分析软件处理肿瘤积分光密度。对胸水体积和积分光密度进行相关性分析，两者之间成线性关系，小鼠的胸水量与对应的积分光密度明显相关（*r*=0.91, *P* < 0.000, 1）（图 7）。

**6:**
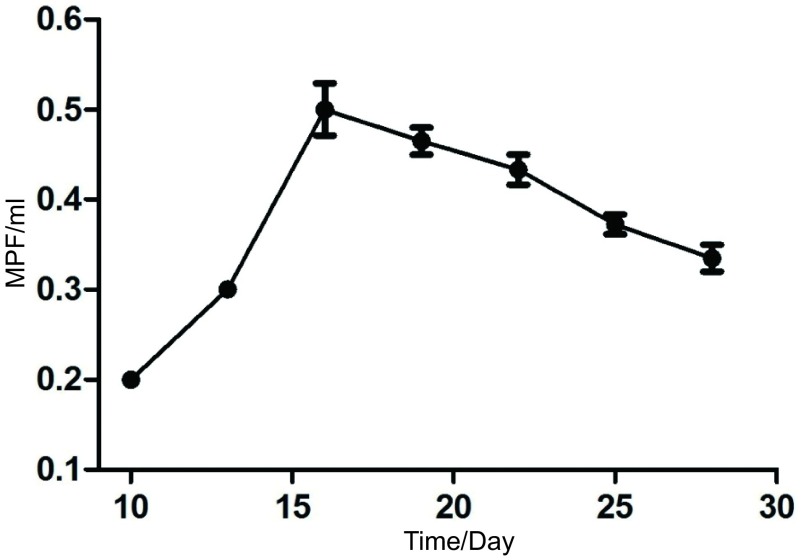
胸腔积液生成曲线：接种后第10天发现血性胸腔积液，量约0.1 mL，随接种时间延长，胸水平均体积逐渐增加，第16天达到高峰0.5 mL，此后维持在0.3 mL-04 mL之间。 The formation curve of malignant pleural effusion. The nude mouse were sacrificed periodically after incubation, bloody pleural effusion was seen at day 10 and the volume was 0.1 mL. Mean volume of pleural fluid increased gradually after inoculation, which reached in a peak at day 16 and the value is 0.5 mL. Since then, it maintained between 0.3 mL-0.4 mL.

**7:**
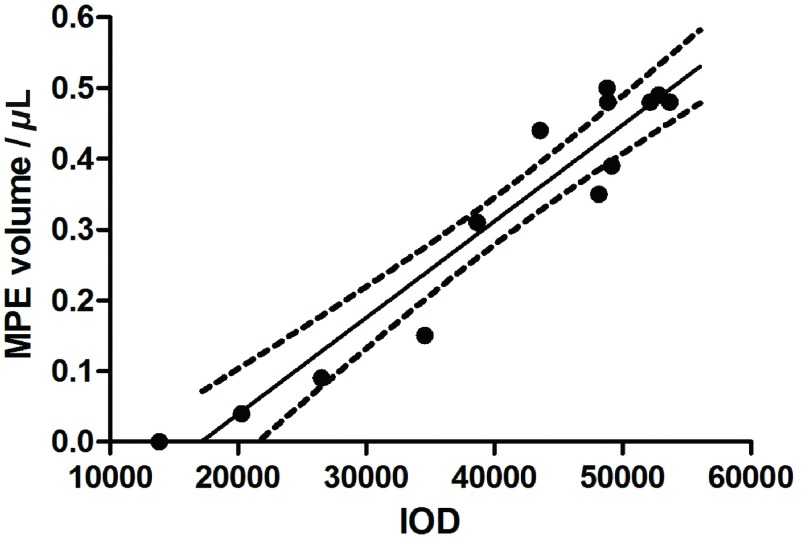
胸水体积和积分光密度之间的相关性分析。小鼠的胸水体积与其积分光密度值明显相关（*r*=0.91, *P* < 0.000, 1）。 Correlation analysis of the pleural fluid volume and the integral optical density after incubation. Significant correlation (*r*=0.91, *P* < 0.000, 1) were observed between pleural fluid volume and integral optical density.

## 3 讨论

1972年Stanton等^[5]^经气道吸入方法建立了MPE模型，此后出现了多种MPE模型，宿主的免疫状态、肿瘤细胞的选择、肿瘤细胞导入胸膜腔的位置和途径是这些模型之间的主要区别。侵袭性强并能分泌血管内皮生长因子的肿瘤细胞是构建MPE的前提^[3]^，近年来多数学者选择经胸腔接种肿瘤细胞直接经胸膜腔接种，虽与肿瘤细胞自发转移到胸膜腔的行为不完全一致，但是可以单纯研究瘤细胞到达胸膜腔的后续行为，包括肿瘤新生血管形成、肿瘤生长、侵袭、转移以及MPE形成等。2005年Stathopoulos^[6]^经胸膜腔接种成功建立MPE模型。此模型在直视下做皮肤、皮下筋膜和肋间肌肉切口，容易损伤肋间血管，小鼠出血多，同时壁层胸膜薄弱，进针后无明显胸膜突破感，不易掌握针尖位置。将靛蓝染料加入细胞悬液中，多次发现蓝色细胞悬液不在胸膜腔内，而在纵隔内或者肺部。此种方法稳定性不高，成瘤率不能保证，动物消耗多，不适合初学者。本次实验借助显微镜进行操作，镜下清晰可见小鼠心脏搏动、双侧肺随呼吸而上下浮动以及肋间血管，肋间隙进针，方向明确。微量移液器有计量精准、针尖圆盾的优点，相对容易控制进针速度，体式显微镜为实验室常用设备，成本低，操作简便，动物损伤相对小，模型稳定，有很好的重复性，成瘤率和成胸水率高。首次将LLC-EGFP细胞接种到胸膜腔内，动态观察到少量肿瘤细胞形成的瘤细胞团块、肿瘤细胞浸润周围组织以及肿瘤细胞向远处转移等一系列过程，与既往的MPE模型相比，本模型通过观察绿色荧光蛋白的表达，能客观评价LLC细胞在裸鼠胸腔内的生长情况，从而能够发现肉眼无法观察到的微小转移灶和淋巴结转移，在进一步探索肿瘤细胞发生胸膜转移的机制的研究中有重要价值。

目前多数文献^[6, 7]^报道在瘤细胞接种后2周内胸部CT可见胸腔积液形成。在病情发展过程中，各时间段胸水生成量及其发生率都在不断变化中。但是目前尚无小鼠MPE模型报道相关数据。本次研究在建模后第7天即行胸部CT检查，并发现2只小鼠有单侧胸腔积液，成胸水率为13%。而在第7天随机解剖小鼠时并未发现胸水，主要与早期成胸水率低有关。随后多只小鼠在同一天发现有血性胸水，平均体积随接种时间逐渐增加，第16天达到峰值，量为0.5 mL。本次实验最高成胸水率为56%。LCC细胞属于鼠源性肺腺癌细胞，与临床上肺腺癌患者终末期并发胸腔积液的几率比较接近。2周后对小鼠行胸部CT检查可见双侧胸腔积液，与文献报道相符合。

IOD为所测结构范围内各像素的光密度值之和，复旦大学肝癌研究所建立的肝癌原位移植模型发现IOD和肿瘤体积相关^[8]^。肿瘤坏死部分不分泌荧光蛋白，故IOD表达不仅与体积相关，还与功能相关，IOD值实质上是有功能的活细胞光密度的总和。本实验对胸腔内肿瘤结节检测IOD值，发现小鼠胸水量与肿瘤IOD值明显相关。这一现象考虑可能与肿瘤新生血管形成有关：肿瘤细胞是代谢活动高度旺盛的细胞，它要持续生长必须有充足的养分供应，故肿瘤必须形成提供自身营养的新生血管^[9]^，由于肿瘤血管的生成过程是一种失去正常控制的无序状态，与正常血管结构相比，肿瘤新生血管的结构缺乏完整性，管壁薄弱，缺乏平滑肌和完整的基底膜，内皮细胞之间存在较大的缝隙，通透性强，血管网结构紊乱，有大量的盲端、动静脉短路以及局部膨出，导致渗出增加及组织间高压，同时也易于癌细胞穿透发生远处转移。此外，肿瘤细胞分泌的一些因子，如血管内皮生长因子（vascular endothelial growth factor, VEGF）等，促进肿瘤微血管发生以及血管壁通透性增加^[10]^。上述机制也是恶性胸腔积液形成的重要原因之一，随着新生血管形成的增加，肿瘤体积逐渐增大，有功能的活细胞代谢旺盛，IOD值呈上升趋势，渗出液生成也随之增加。胸水量和IOD值随接种时间延长逐渐增加，而到终末期，肿瘤组织缺血、破溃、坏死，IOD和胸水体积不再增加。在同一只小鼠，IOD和胸水量之间的变化，以新生血管为纽带呈现一定的相关性。Stathopoulos^[6]^发现胸膜上肿瘤结节数量与胸水量有相关性（*P* < 0.005）。在恶性胸腔积液的抗血管生成治疗中，通过检测IOD值的变化，可间接反映胸水体积的变化趋势，有助于疗效评价。由于本实验裸鼠数量有限，需要增大样本量，进一步探讨该实验现象。

荧光体视镜未发现肿瘤侵及肺部、头颅、骨骼等常见肺癌转移部位，考虑与荧光成像的特性有关，由于生物的自体荧光及组织光吸收散射作用的影响，荧光成像的最高灵敏度也只能检测到约1×10^6^个细胞^[11]^。另外，能否在活体检测GFP肿瘤细胞的转移，除了需要转移的肿瘤细胞达到一定数量外，与转移灶在体内的位置密切相关。胸腔内转移瘤体积小，又受到气体和皮肤的干扰，在体外活体观察荧光的强度大幅度减弱。在预实验中经可逆性皮瓣可观察到局部范围的绿色荧光，但远处微小转移灶显像不清晰。皮瓣可扩大欲检测器官的荧光通路，我们经可逆性皮瓣成功观察到裸鼠肺癌原位移植瘤的生长和转移^[12]^，但是对胸腔内多发、微小转移灶，皮瓣不能提供足够的荧光通路，此外，胸腔内肿瘤生长和转移较快，频繁打开皮瓣对裸鼠损伤较大，部分裸鼠尚未观察到远处转移时已经死亡，只能将裸鼠处死并打开胸腔进行观察。据文献^[13]^报道，目前已发现的最亮的荧光蛋白是一种叫Katushka的红色荧光蛋白，发射波长超过620 nm，能减少组织吸收和散射，具有快速成熟、高pH稳定性和光稳定性的特点，可对深部肿瘤、转移性淋巴结等进行活体观察。将来可能会使胸腔内转移瘤的活体、非侵入观察成为现实。

综上所述，本实验用经胸膜腔注射方法，首次建立了表达绿色荧光蛋白的胸腔积液模型，通过观察绿色荧光蛋白的表达和胸腔积液的形成特征，探索了肺癌细胞侵袭胸膜、转移的特征，以及胸腔积液与肿瘤荧光面积、IOD值之间的关系，实时模拟了晚期肺癌侵袭胸膜的一系列生物学行为，为探索药物治疗肺癌合并恶性胸腔积液提供了科学、合理的实验研究平台。
